# The complete mitochondrial genome of *Reticulitermes periflaviceps* (Isoptera: Rhinotermitidae)

**DOI:** 10.1080/23802359.2019.1688701

**Published:** 2019-11-14

**Authors:** Xin Ye, Yin Hu, Haihong Chen, Yiyuan Liao, Tong Chen, Dayu Zhang

**Affiliations:** a College of Agricultural and Food Science, Zhejiang A & F University, Hangzhou, P. R. China;; b National Termite Control Center, Hangzhou, P. R. China;; c Ningbo Housing Safety Management and Service Center, Ningbo, P. R. China

**Keywords:** *Reticulitermes periflaviceps*, Isoptera, mitochondrial genome

## Abstract

The mitochondrial genome of the *Reticulitermes periflaviceps* contains 15,925 bp of nucleotide in length including 13 protein-coding genes, 22 transport RNAs, 2 ribosomal RNAs, and a non-coding region. The overall A + T among the genome sequence is 65.64%. The start codons of all protein-coding genes are ATN and the stop codon is TAA except TAG for Nad1 and incomplete T for COII and Nad5. The lengths of 12sRNA and 16sRNA genes are 743 and 1309 bp, respectively, and the control region was 1118 bp in length, with two repeating tandem regions. The phylogenetic tree revealed that *R. periflaviceps* and *Reticulitermes kanmonensis* constituted a sister group to *Reticulitermes flaviceps*. The mitochondrial genome here provides a resource for evolution analysis within termites especially *Reticulitermes*.

*Reticulitermes* is the most abundant genus of lower termites with a small population and relatively scattered nests (Cameron and Whiting 2007; Liu et al. 2019). This group of termites can cause severe damages to household structures and trees. *Reticulitermes* is often considered to be a transitional group between lower termites and higher termites. *Reticulitermes periflaviceps* was first discovered in Guangdong, China, This species has spread to many provinces in China (Huang et al. 2000). In this study, the complete mitochondrial genome sequence of *R. periflaviceps* is reported for the first time. The sequence information can be a basis for molecular classification and evolution analysis of the termites.

Specimens were collected from Ningbo, China and kept in the insect lab at Zhejiang A & F University with accession number NB0067-ZH-16. The complete circular mitochondrial genome of *R. periflaviceps* has a length of 15,925 bp and encodes 37 genes including 13 protein-coding genes (*PCGs, nad1-6, nad4L, atp6, atp8, cox1-3, and cytb*), 22 transfer RNA (tRNA), 2 ribosomal RNA (rRNA), and a non-coding control region (D-loop). Most of these PCGs are located on the H-strand except for nad1, nad4, nad4l, nad5, and 8 tRNA genes (*trnQ, trnC, trnY, trnF, trnH, trnP, trnL1, and trnV*). The genetic compositions and coding sequences are similar to other termites (Legendre et al. 2008; Vargo and Husseneder 2009; Liu et al. 2019).

The base composition of *R. periflaviceps* shows that the percentage of A and T (65.64%) is much higher than that of G and C (34.36%). The mitochondrial genome composition of *R. periflaviceps* includes intergenic spacers and overlapping regions. The intergenic spacers contain 18 regions with a total length of 118 bp, and the overlapping ranging in size from 1 to 8 bp on 6 regions.

The *R. periflaviceps* protein-coding region is 11,166 bp in length. The start codons of all protein-coding genes are ATN, COI starts with ATT, ATP6, ATP8, and Nad3 start with ATA, and the rest of the protein genes start with ATG. Except for Nad1 with TAG, COII, and Nad5 with incomplete T as stop codon, the remaining protein genes all adopt TAA as stop codon.

There are 22 tRNA genes in the mitochondrial genome of *R. periflaviceps*. Except for the tRNA-Ser, which lacks the dihydrorubamide (DHU) arm, the other tRNAs have the Classical cloverleaf structures. The 12sRNA and 16sRNA gene (*rrnS* and *rrnL*) of *R. periflaviceps* is 74 bp and 1309 bp in length, respectively.

The control region (D-loop) with 1118 bp in size is located between *rrnS* and *trnI* gene. The D-loop region contains two repeating regions in length of 184 and 588 bp, respectively. The distribution patterns are consistent with other *Reticulitermes* termites (Cameron and Whiting 2007; Liu et al. 2019).

Using a maximum likelihood method (Guindon et al. 2010), a phylogenetic tree among *Reticulitermes* was constructed from a dataset that contained all the nucleotide sequences of PCGs of *Reticulitermes* mitochondrial genes. *Incisitermes minor and Mastotermes darwiniensis* were set as outgroups (Figure 1). The tree revealed that *R. periflaviceps* was closest to *Reticulitermes kanmonensis* and constituted a sister group to *Reticulitermes flaviceps*.

**Figure 1.**
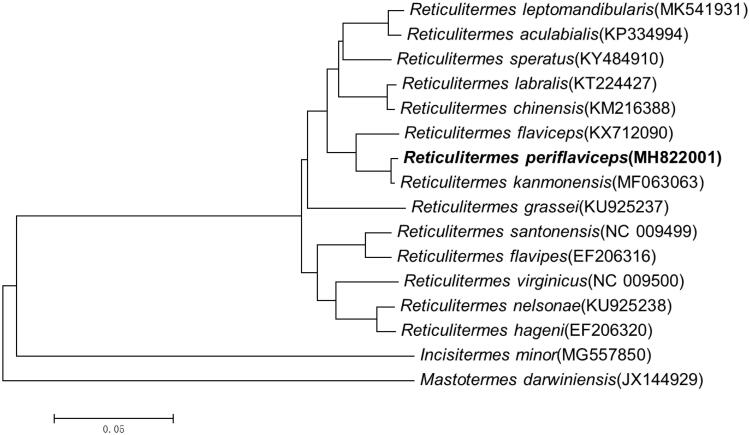
The phylogenetic tree was built using the nucleotide sequences of 13 PCGs. *Incisitermes minor* and *Mastotermes darwiniensis* were set as outgroups. Leaf names were presented as species names and Genbank accession number.

## Nucleotide sequence accession number

The complete genome sequence of *R. periflaviceps* has been assigned GenBank accession number (MH822001).
